# Case Report: Late diagnosis of McCune-Albright with severe kyphoscoliosis, acromegaly and tertiary hyperparathyroidism

**DOI:** 10.3389/fendo.2025.1464945

**Published:** 2025-04-28

**Authors:** Anna Bogusławska, Maria Komisarz-Calik, Alicja Hubalewska-Dydejczyk, Márta Korbonits, Pauline Romanet, Anne Barlier, Aleksandra Gilis-Januszewska

**Affiliations:** ^1^ Chair and Department of Endocrinology, Jagiellonian University, Medical College, Cracow, Poland; ^2^ Centre for Endocrinology, William Harvey Research Institute, Barts and the London School of Medicine and Dentistry, Queen Mary University of London, London, United Kingdom; ^3^ Aix Marseille Univ, Assistance Publique-Hôpitaux De Marseille (APHM), Institut national de la santé et de la recherche médicale (INSERM), Marseille Medical Genetics (MMG), La Timone Hospital, Laboratory of Molecular Biology GEnOPe, Biogenopole, Marseille, France

**Keywords:** McCune-Albright syndrome, acromegaly, tertiary hyperparathyroidism, GNAS gene, fibrous dysplasia (FD)

## Abstract

McCune–Albright syndrome (MAS) is a rare genetic disorder caused by somatic activating variants of the GNAS gene. Due to the mosaic state of the variants, the clinical presentation of MAS varies widely depending on the tissues involved. We present a case of a 40-year-old woman who was admitted to the Pulmonary Unit due to progressive pulmonary insufficiency secondary to severe scoliosis. Upon physical examination, hyperpigmented skin lesions on the neck, features of acromegaly, and scoliosis were noted. Radiographic imaging revealed osteolytic lesions of the axial skeleton, which were suspected to be metastases. Imaging via 18F-fluorodeoxyglucose positron emission tomography (18F-FDG PET) did not confirm metastases and revealed a pituitary lesion. The laboratory workup confirmed acromegaly. Additionally, hypercalcemia, normophosphatemia, elevated parathyroid hormone level, and decreased urine calcium excretion were found. Further examinations revealed kidney stones, cholecystolithiasis, and severe osteoporosis. During follow-up visits, hypophosphatemia has been observed. Bone scintigraphy revealed increased tracer uptake in multiple skeletal system parts, corresponding to degenerative changes. Genetic testing using Sanger sequencing was negative for *MEN1* and *CDKN1B* mutations but revealed a common germline, heterozygous *GNAS* variant NM_000516.7:c.531-13_531-10del (rs576071932) – classified as a variant of uncertain significance (RCV000597562.1) with a minor allele frequency of 0.265%. Digital Droplet Polymerase Chain Reaction in the circulating cell-free DNA was negative for R201C and R201H GNAS mutation. This case emphasizes that acromegaly, skeletal deformity, hyperpigmented skin lesions, and hyperfunction of the thyroid and parathyroid glands may lead to suspicion of MAS. The diagnosis is often made clinically based on two or more characteristic symptoms. Genetic confirmation of MAS can be challenging.

## Introduction

McCune–Albright syndrome (MAS) is a very rare genetic disease with a prevalence between 1:100 000 and 1:1 000 000 (1). It is caused by somatic activating variants of guanine nucleotide binding protein, alpha stimulating (*GNAS)* gene (2). Classically, MAS is characterized by the presence of a triad of polycystic fibrous dysplasia (FD), precocious puberty, and café-au-lait spots (1, 3). However, due to somatic mosaicism, the phenotype of patients could be variable in extent and severity. Mutations of *GNAS* result in constant activation of adenylate cyclase and lead to autonomous hyperfunction of affected tissues (4). Growth hormone excess affects approximately 20% of cases, mostly due to somatotroph pituitary neuroendocrine tumors (PitNET) or pituitary hyperplasia (5). Diagnosis of MAS is usually established based on clinical features (2, 6, 7). Molecular diagnosis is possible but is not routinely available. Additionally, biological samples bearing a low level of mosaicism frequently lead to false-negative results with an underestimation of causative molecular alterations when using Sanger sequencing (5, 8).

Here, we present the case of a 47-year-old female patient with an unusual presentation of previously undiagnosed MAS with acromegaly, subclinical hyperthyroidism, tertiary hyperparathyroidism, café-au-lait spots, and diffuse spinal FD since childhood, resulting in thoraco-lumbar scoliosis and secondary pulmonary insufficiency.

## Case description

**Figure 1:**
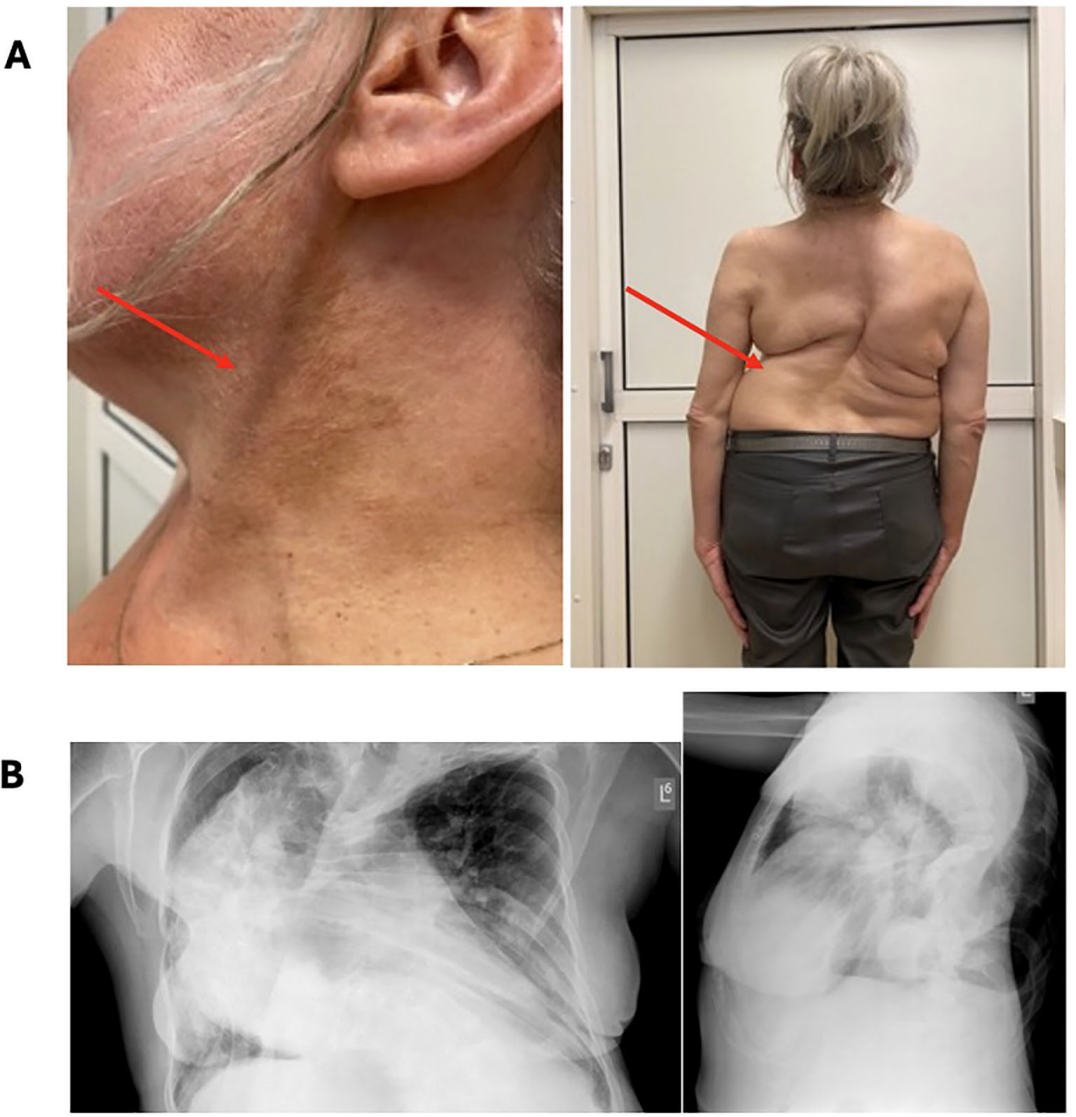
Clinical presentation of the patient: **(A)** café-au-lait macules, **(B)** severe bone deformity of the vertebrae causing pulmonary insufficiency **(B)**.

**Figure 2:**
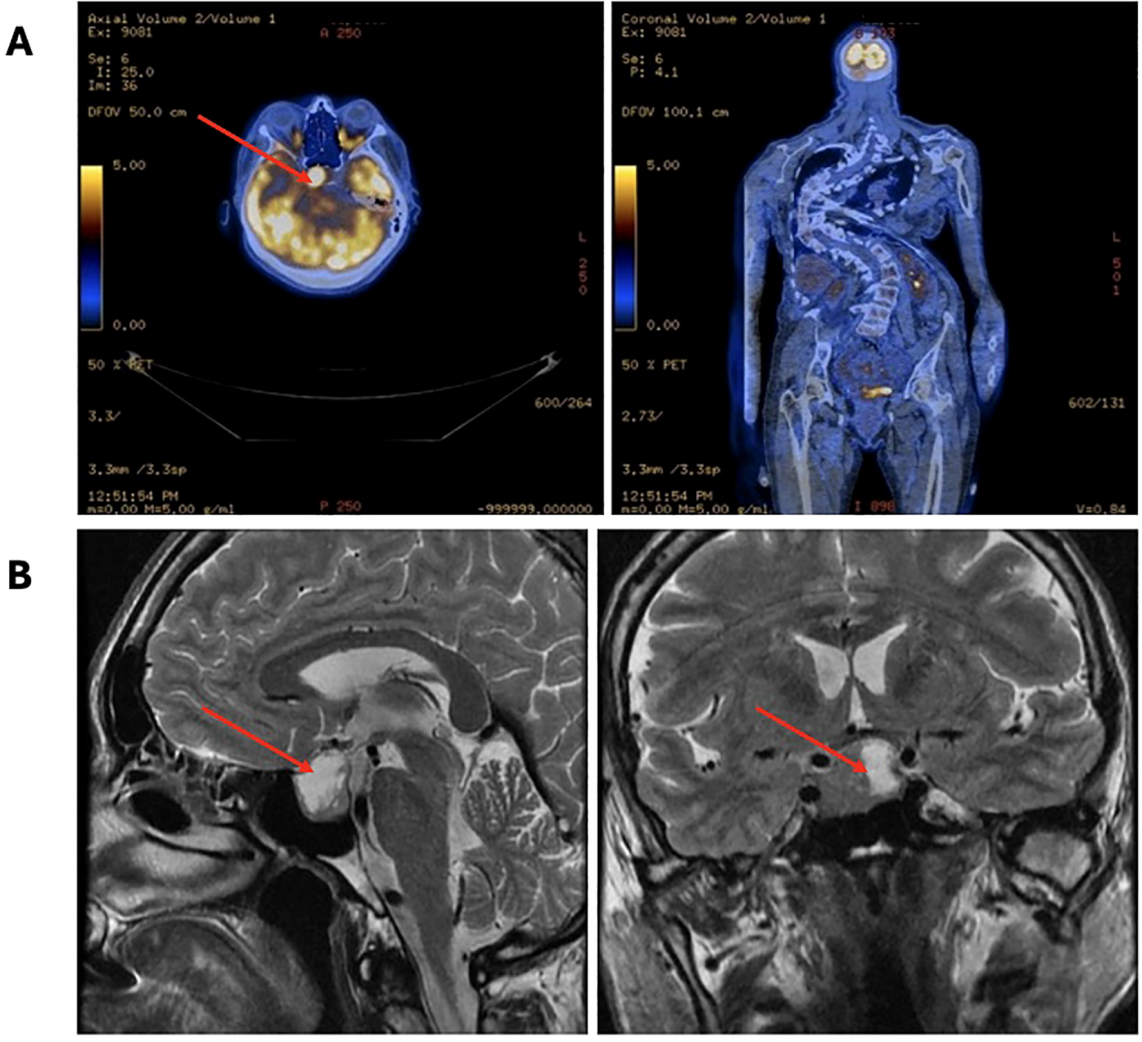
X-ray showing osteolytic lesions in the skeletal system, initially suspected to be metastatic in nature.

Our patient was originally admitted to the Pulmonary Unit at the age of 40 years due to exacerbation of dyspnea (January 2018). She suffered from long-lasting severe progressive pulmonary insufficiency and heart failure (NYHA I/II) secondary to severe scoliosis (**Figure 1**) and was treated at home with mechanical noninvasive ventilation (NIV) since 2001. A routine chest X-ray revealed osteolytic lesions in the skeletal system, which were initially suspected to be metastatic. Positron emission tomography with 2-deoxy-2-[fluorine-18]fluoro-D-glucose integrated with computed tomography (18F-FDG PET/CT) did not confirm a neoplastic process; however, increased tracer accumulation was noted in the sellar region (**Figure 2A**). The patient was then transferred to the Endocrinology Department. Hyperpigmented skin lesions on the neck, severe progressive scoliosis since childhood (**Figure 1**), umbilical hernia, enlarged feet and hands, acromegalic facial changes, and surprising normal height (final height 163 cm, midparental height 164.5 cm) were noted, suggesting that without the presence of significant scoliosis, her height could have been significantly greater than the predicted adult height. A pituitary MRI (April 2018) revealed a pituitary tumor with dimensions of 27.5x18.5x23.0 mm extending to the right cavernous sinus (**Figure 2B**). Laboratory workup confirmed acromegaly with concomitant hyperprolactinemia (IGF-1 level was 1.5x the upper limit of normal range (NR)). Other findings were as follows: random growth hormone (GH) level was 5.48 ug/L, nadir GH on oral glucose tolerance test (OGTT) was 5.48 ug/L and prolactin level was 2x above the upper limit of NR. Preoperatively, a long-acting somatostatin analogue was introduced (octreotide LAR 30 mg) which did not decrease the level of IGF-1. After 6 months of treatment, the patient underwent transsphenoidal surgery in 2019. Histopathological examination revealed a mixed tumor with sparsely granulated somatotroph pituitary neuroendocrine tumor (PitNET)/immature PIT1-lineage along with immunostaining findings of GH+, PRL+, TSH-, ACTH-, LH-, FSH-, 1% Ki-67, chromogranin+, p53, synaptophysin+, SF1-, Tpit-, PIT1+ and GATA3-. After surgery, biochemical control of acromegaly improved with IGF-1 and prolactin within the normal range and OGTT GH nadir of 0.6 ug/L. In MRI, a residual pituitary tissue was observed.

**Figure 3:**
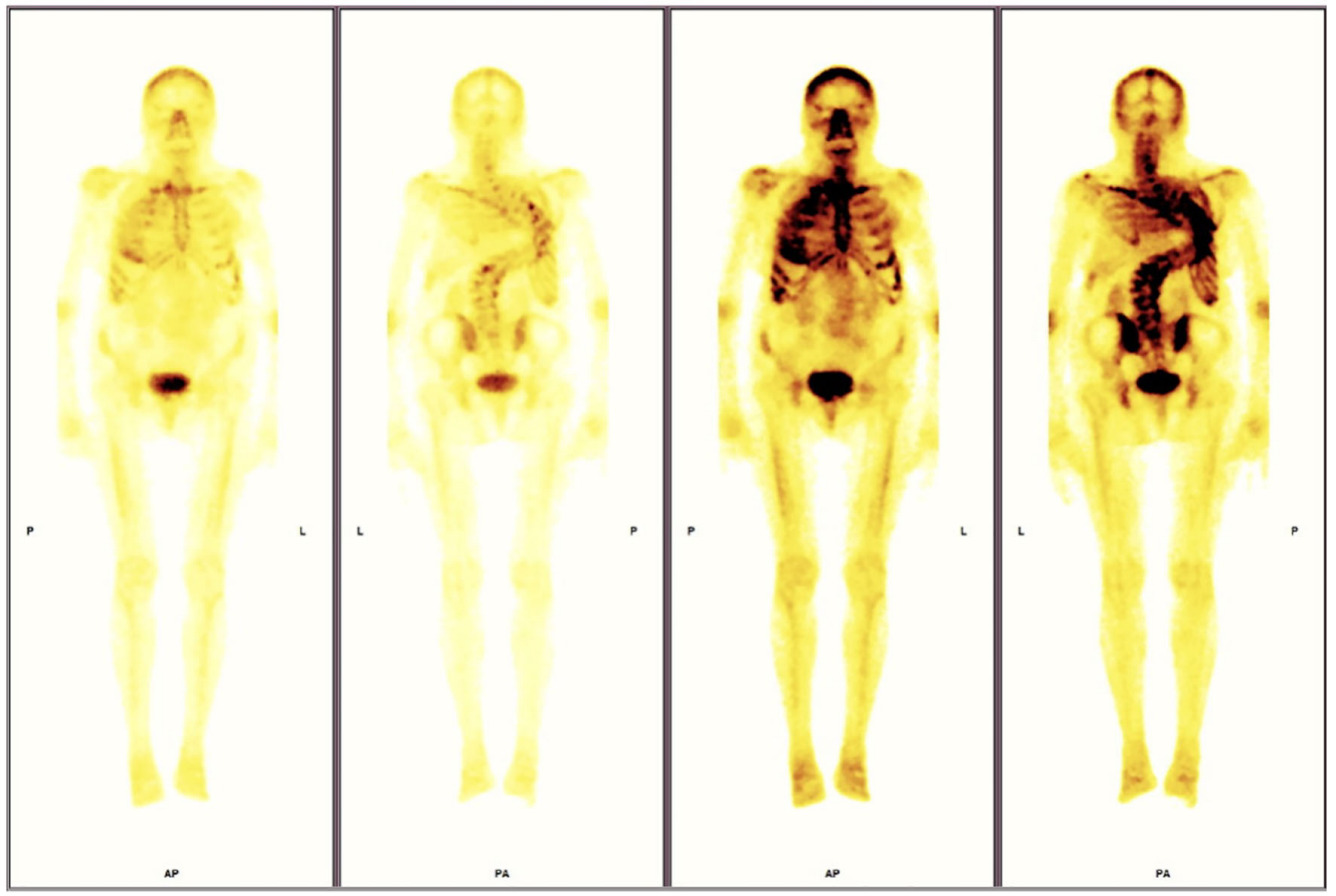
**(A)** 18F-FDG PET/CT images revealed increased tracer accumulation in the sellar region. **(B)** Pituitary MRI images of the pituitary fossa showing a tumor (27.5x18.5x23.0 mm).

In 2018, mild hypercalcemia (2.63 mmol/l, NR 2.15-2.55 mmol/l), normophosphatemia (1.18 mmol/l, NR 0.81-1.45 mmol/l) with elevated parathyroid hormone level (105.9 pg/mL, NR 14.90–56.90 pg/mL), and decreased urine calcium excretion (2.45 mmol/24 h) were noted. Further examinations revealed kidney stones, cholecystolithiasis, and severe osteoporosis. Fibroblast growth factor 23 (FGF23) was measured, and the values were elevated (126 kRU/l, NR 26-110 KRU/l). Imaging via 99mTc-MIBI single-photon emission computed tomography/computed tomography (SPECT/CT) of the parathyroid glands did not reveal typical focal accumulation of the tracer (initially in 2019 and repeated in 2022). Hypophosphatemia has been observed with urine excretion of phosphorus (21.1 mmol/24h) close to the upper limit of normal. Bone scintigraphy in 2022 revealed increased tracer uptake in the axial skeletal system, shoulders, sternoclavicular joints and hips, which could indicate either degenerative changes or could correspond to the characteristic changes of FD (**Figure 3**). Imaging via CT and MRI did not reveal the involvement of FD at the base of the skull. One region in the ethmoid sinus was suspected to be FD; however, a comparison with preoperative CT and postoperative MRI showed inflammation in this area.

Thyroid ultrasound revealed a goiter (volume 32 ml) and a single hypoechogenic lesion (8 mm). Biochemical analysis revealed subclinical hyperthyroidism with negative TSH receptor antibodies (TRAB), positive anti-thyroid peroxidase (ATPO) and anti-thyroglobulin antibodies (ATG). Menarche was delayed, with the first menstrual period observed at the age of 20 years; however, regular menstrual cycles followed thereafter. Additionally, there was no history of neonatal hypercortisolism.

In the most recent follow-up, the patient had a calcium level of 2.79 mmol/l, hypophosphatemia (0.77 mmol/l), elevated parathyroid hormone level (87.9 pg/mL), and normal urine excretion of calcium and phosphate. Without any treatment, her thyroid hormone levels were within NR (TSH 0.94 uIU/ml, fT3 4.56 pmol/l, fT4 15.4 pmol/l).

Genetic testing using Sanger sequencing was negative for *MEN1* and *CDKN1B* mutations in blood-derived DNA, but revealed a common splice site variant in *GNAS* (heterozygous NM_000516.7:c.531-13_531-10del, rs576071932), representing a deletion of four nucleotides, localized in intron 6 of *GNAS*. The NCBI ClinVar database classified this as a variant of uncertain significance (RCV000597562.1) with a frequency of 0.265% in European non-Finnish populations (GnomAD database). Digital Droplet Polymerase Chain Reaction in the circulating cell-free DNA was negative for R201C and R201H *GNAS* mutations. Further genetic evaluation of DNA extracted from formalin-fixed paraffin-embedded pituitary tissue was negative for the classical *GNAS* mutations related to MAS (Sanger sequencing).

## Discussion

The most prominent clinical feature of this patient was severe kyphoscoliosis since childhood which significantly affected her quality of life. Fibrous dysplasia has a broad clinical spectrum, ranging from incidentally diagnosed radiographic findings to severe and disabling disease. In our patient, the chest deformity led to severe cardiovascular and pulmonological complications. Fibrous dysplasia of the bone is the major manifestation of MAS (seen in 98% of patients), followed by café-au-lait spots. The most commonly involved skeletal regions are the proximal femur and base of the skull, but the real prevalence of spinal lesions and any association with scoliosis in FD/MAS could be underestimated (9, 10). When GH excess is diagnosed in MAS patients it is typically associated with FD of the skull base, which was not observed in our patient (5).

In our patient, mild hypercalcemia with progressive hypophosphatemia with elevated PTH levels was noted; however, she did not have increased urinary calcium secretion, otherwise characteristic of primary hyperparathyroidism. In MAS, the replacement of bone marrow with fibrous stroma is observed (11, 12). An abnormal fibroblastic bone phenotype results in the overproduction of FGF23, which decreases renal reabsorption of phosphate (13, 14). In our patient, tertiary hyperparathyroidism was observed in the course of long-standing secondary hyperparathyroidism due to FD-related FGF23-mediated hypophosphatemia. Additionally, hypophosphatemia likely contributed to severe, inoperable scoliosis. Burosumab, a humanized monoclonal antibody for FGF23 which will increase renal tubular reabsorption of phosphate represents a promising treatment option for hypophosphatemic osteomalacia in McCune-Albright syndrome, however further clinical trials are needed to establish standardized protocols.

At the time of diagnosis, subclinical hyperthyroidism was noted. An additional autoimmune component (positive ATG, ATPO antibodies, but genitive TRAB) was also observed with an enlarged thyroid gland (volume 32 ml, NR 18 ml). Hyperthyroidism is the second most common manifestation of MAS as a result of persistent activation of the stimulatory G protein. This can lead to thyroid hyperplasia, overactive thyroid function, and increased deiodinase activity (15). The measurement of free T3/T4 ratios might be helpful, with a ratio of >20 being suggestive of the disease (2); however, in our patient, this ratio was much lower. In MAS patients, diffuse heterogeneity and hyper- or hypoechoic lesions can be present on thyroid ultrasonographic examination. Hyperthyroidism is often subclinical without overt symptoms; however, increased thyroid morbidity among patients has been observed. In the most recent biochemical and clinical assessment, our patient had no signs of thyrotoxicosis, and we continue to monitor her with periodic follow-up visits. Subclinical hyperthyroidism in this patient is probably related to the disease.

Hyperthyroidism in McCune Albright patients is usually resistant to antithyroid drugs, making radioactive iodine therapy and surgery the most effective long-term solution (2).

McCune–Albright syndrome-associated acromegaly is usually diagnosed during early adulthood. In our patient, PitNET was incidentally discovered at the age of 40 years. She presented with enlarged feet and hands, acromegalic facial changes, and metabolic complications such as glucose intolerance.

Growth hormone excess affects one-third of patients, with concomitant hyperprolactinemia seen in 81% of cases (5). This was also observed in our patient. Pituitary surgery was the first-line treatment in our patient due to the absence of FD involvement of the skull base. Postoperatively, biochemical control of acromegaly was achieved. In MAS patients, remission of acromegaly due to diffuse pituitary disease is observed in only 12% of patients after surgical treatment. In some cases, total hypophysectomy should be considered. However, if neurosurgery is contraindicated, somatostatin analogues are recommended. In resistant cases, pegvisomant monotherapy or in combination with somatostatin analogues should be offered (5, 16). Radiotherapy is generally not recommended for fibrous dysplasia in MAS patient due to the increased risk of the development of osteosarcoma (2).

Skin manifestations of MAS include café-au-lait macules and could be the first symptom of this disease, with presentation shortly after birth. These hyperpigmented skin lesions are characterized by jagged, irregular borders, and they typically do not cross the midline of the body (17). Our patient presented with bilateral café-au-lait spots on the neck typical for MAS-like lesions.

The occurrence of acromegaly and symptomatic hypercalcemia should raise a suspicion for syndromic disease (18). Genetic testing was negative for *MEN1* and *CDKN1b* mutations. Additional endocrinopathies (subclinical hyperthyroidism in our patient) and involvement of the skin and the skeletal system resulting in severe kyphoscoliosis should prompt the diagnosis of MAS.

McCune–Albright syndrome is a hereditary disease with *de novo* mutations. The mutation in *GNAS* occurs during the early postzygotic stage and results in somatic mosaicism. The diagnosis of MAS is a clinical diagnosis based on two or more characteristic symptoms, with the exact phenotype being dependent on the affected tissues. Mutation in *GNAS* may help confirm the diagnosis; however, genetic testing is challenging due to variable levels of mosaicism in the affected tissue. Low-level mosaicism may be undetectable using Sanger sequencing; however, even more reliable methods, such as droplet digital polymerase chain reaction, are positive only in 58.3% of FD/MAS-suspected patients from whole blood DNA and in 80% of FD/MAS-suspected patients from circulating cell-free DNA (19). In our patient, *GNAS* mutation associated with MAS was negative in Sanger sequencing in DNA from peripheral blood leukocytes and DNA from the pituitary tissue as well as in Digital Droplet Polymerase Chain Reaction in whole blood DNA and Circulating Cell-Free DNA. PCR amplification of samples from affected tissue has an approximate sensitivity of 80-90%, while the approximate sensitivity from peripheral blood lymphocytes is 20-30% (20).

## Conclusions

Acromegaly, severe scoliosis, hyperpigmented skin lesions, and hyperfunction of the thyroid and parathyroid glands should raise suspicion for MAS. Acromegaly can occur in MAS with an absence of FD in the skull base. The variable clinical and biochemical presentations can make the diagnosis of MAS difficult, which might have a diverse clinical course including tertiary hyperparathyroidism due to long-standing secondary hyperparathyroidism in the course of FD-related, hypophosphatemia. Genetic confirmation of this rare syndrome can be challenging; however, a negative result does not rule out MAS.

## Data Availability

The datasets presented in this study can be found in online repositories. The names of the repository/repositories and accession number(s) can be found in the article/supplementary material.

## References

[1] Dumitrescu CE Collins MT. McCune-Albright syndrome. Orphanet J Rare Dis. (2008) 3:12. doi: 10.1186/1750-1172-3-12 18489744 PMC2459161

[2] Javaid MK Boyce A Appelman-Dijkstra N Ong J Defabianis P Offian A. Best practice management guidelines for fibrous dysplasia/McCune-Albright syndrome : a consensus statement from the FD/MAS international consortium. Orphanet J Rare Dis. (2019) 14:267. doi: 10.1186/s13023-019-1255-6 31752972 PMC6868680

[3] Albright F Butler AM Hampton AO Smith P. Disseminata, areas of pigmentation and endocrine. N Engl J Med. (1937) 216:721–46.

[4] Weinstein LS Shenker A Friedman E Spiegel AM Gejman PV Merino MJ. Activating mutations of the stimulatory g protein in the mcCune–albright syndrome. N Engl J Med. (1991) 325:1688–95. doi: 10.1056/NEJM199112123252403 1944469

[5] Salenave S Boyce AM Collins MT Chanson P. Acromegaly and McCune-Albright syndrome. J Clin Endocrinol Metab. (2014) 99:1955–69. doi: 10.1210/jc.2013-3826 PMC4037730 24517150

[6] Narumi S Matsuo K Ishii T Tanahashi Y Hasegawa T. Quantitative and sensitive detection of GNAS mutations causing McCune-Albright syndrome with next generation sequencing. PloS One. (2013) 8:e60525. doi: 10.1371/journal.pone.0060525 23536913 PMC3607597

[7] Lumbroso S Paris F Sultan C. McCune-Albright syndrome: Molecular genetics. J Pediatr Endocrinol Metab. (2002) 15:875–82. 12199345

[8] Elli FM de Sanctis L Bergallo M Maffini MA Pirelli A Galliano I. Improved molecular diagnosis of McCune–Albright syndrome and bone fibrous dysplasia by digital PCR. Front Genet. (2019) 10:862. doi: 10.3389/fgene.2019.00862 31620168 PMC6760069

[9] Leet AI Magur E Lee JS Wientroub S Robey PG Collins MT. Fibrous dysplasia in the spine: prevalence of lesions and association with scoliosis. J Bone Jt Surg. (2004) 86:531–7. doi: 10.2106/00004623-200403000-00011 14996879

[10] Michev A Lungarotti L Prevedoni Gorone MS Apicella A Di Vincenzo G Marseglia GL. Scoliosis with peculiar radiological features in a patient with McCune-Albright syndrome. Clin Case Reports. (2021) 9:e04242. doi: 10.1002/ccr3.4242 PMC8301555 34322238

[11] Lavi-Moshayoff V Wasserman G Meir T Silver J Naveh-Many T. PTH increases FGF23 gene expression and mediates the high-FGF23 levels of experimental kidney failure: A bone parathyroid feedback loop. Am J Physiol - Ren Physiol. (2010) 299:882–9. doi: 10.1152/ajprenal.00360.2010 20685823

[12] Liu S Tang W Zhou J Stubbs JR Luo Q Pi M. Fibroblast growth factor 23 is a counter-regulatory phosphaturic hormone for vitamin D. J Am Soc Nephrol. (2006) 17:1305–15. doi: 10.1681/ASN.2005111185 16597685

[13] De Castro LF Ovejero D Boyce AM. Diagnosis of endocrine disease: Mosaic disorders of FGF23 excess: Fibrous dysplasia/McCune-Albright syndrome and cutaneous skeletal hypophosphatemia syndrome. Eur J Endocrinol. (2020) 182:R83–99. doi: 10.1530/EJE-19-0969 PMC7104564 32069220

[14] Furlanetto TW. Etiology of hyperparathyroidism in McCune-Albright syndrome. J Maxillofac Surg. (2009) 67:2037. doi: 10.1016/j.joms.2009.04.007 19686948

[15] Celi FS Coppotelli G Chidakel A Kelly M Brillante BA Shawker T. The role of type 1 and type 2 5′-deiodinase in the pathophysiology of the 3,5,3′-triiodothyronine toxicosis of McCune-Albright syndrome. J Clin Endocrinol Metab. (2008) 93:2383–9. doi: 10.1210/jc.2007-2237 PMC2435649 18349068

[16] Wong SC Zacharin M. Long-term health outcomes of adults with McCune- Albright syndrome. Clin Endocrinol (Oxf). (2017) 87:627–34. doi: 10.1111/cen.13419 28699175

[17] Benedict PH Szabó G Fitzpatrick TB Sinesi SJ. Melanotic macules in Albright’s syndrome and in neurofibromatosis. JAMA J Am Med Assoc. (1968) 205:618–26. doi: 10.1001/jama.1968.03140350028006 4969843

[18] Bogusławska A Korbonits M. Genetics of acromegaly and gigantism. J Clin Med. (2021) 10:1–24. doi: 10.3390/jcm10071377 PMC8036715 33805450

[19] Romanet P Philibert P Fina F Cuny T Roche C Ouafik L. Using digital droplet polymerase chain reaction to detect the mosaic GNAS mutations in whole blood DNA or circulating cell-free DNA in fibrous dysplasia and McCune-Albright syndrome. J Pediatr. (2019) 205:281–285.e4. doi: 10.1016/j.jpeds.2018.09.070 30442414

[20] Boyce AM Florenzano P de Castro LF Collins MT. Fibrous dysplasia/McCune-Albright syndrome summary. GeneReviews. (2019) 1:1–40.

